# PTA-DFS study: design of a randomised controlled trial assessing the effects of early percutaneous transluminal angioplasty on the healing of diabetic foot ulcers in persons with type 2 diabetes

**DOI:** 10.1186/s12872-025-05288-1

**Published:** 2025-11-14

**Authors:** Kálmán B. Bódis, David-Ioan Florea, Shouheng Goh, Nicolas Kramser, Tobias Wienemann, Christian Binsch, Manuel Stern, Klaus Pfeffer, Malte Kelm, Michael Roden, Robert Wagner, Lucas Busch, Patricia Wischmann, PTA-DFS Study Group, Kálmán B. Bódis, Patricia Wischmann

**Affiliations:** 1 Department of Endocrinology and Diabetology, Medical Faculty and University Hospital Düsseldorf, Heinrich Heine University, Düsseldorf, Germany; 2 Institute for Clinical Diabetology, German Diabetes Center, Leibniz Institute for Diabetes Research at Heinrich Heine University, Düsseldorf, Germany; 3 German Center for Diabetes Research (DZD E.V.), Partner Düsseldorf, München-Neuherberg, Germany; 4 Department of Cardiology, Pulmonology, and Vascular Medicine, Medical Faculty, Heinrich Heine University of Düsseldorf, Düsseldorf, Germany; 5 Cardiovascular Research Institute Düsseldorf (CARID), Medical Faculty and University Hospital Düsseldorf, Heinrich Heine University, Düsseldorf, Germany; 6 Institute of Medical Microbiology and Hospital Hygiene, Medical Faculty and University Hospital Düsseldorf, Heinrich Heine University, Düsseldorf, Germany

**Keywords:** Diabetic foot syndrome, Peripheral artery disease, Diabetic foot ulcers, Wound microbiome, Percutaneous transluminal angioplasty

## Abstract

**Background:**

Peripheral arterial disease (PAD) and local infections increase the risk of non-healing diabetic foot ulcers (DFU) and limb amputations but are treatable by percutaneous transluminal angioplasty (PTA), local wound care and antibiotic therapy. The exact time to treat chronic leg artery stenosis (LAS) and the role of microbiome composition in DFU remain unclear. This study aims to assess whether an early PTA within 48 h after diagnosing a LAS offers advantages over standard care.

**Methods:**

The PTA-DFS Study is a randomised controlled monocentric trial including individuals with T2D and DFU, aged > 18 years with haemodynamically relevant chronic LAS. The primary study endpoint is to investigate the impact of the early PTA within 48 h on wound-healing assessed by wound area changes after PTA using a 3D-camera with artificial intelligence (AI)-based wound-analysis-system. The secondary endpoint is the effect of early PTA on the combined occurrence of major adverse limb (MALE) and safety-related cardiac events (MACE) over 12 month post-angioplasty using time-to-event analysis. Additional secondary outcomes are time to complete wound healing, major amputation rate and the need for new revascularization. Explanatory variables for wound healing are wound microbiome changes using whole-genome sequencing and oxygen saturation of the wound environment measured using near-infrared spectroscopy. Data will be collected at baseline, 24 h, 1, 2, 3, 6, and 12 months after PTA. Diabetic kidney disease, distal symmetric polyneuropathy, retinopathy, cardiomyopathy, LAS will be assessed by laboratory analyses, clinical scores, AI-based fundus photography, echocardiography, duplex sonography, and pulse oscillography.

**Discussion:**

The PTA-DFS aims to improve diagnostic and therapeutic algorithms, risk assessment and enable tailored therapies for persons with T2D and ischemic DFU.

**Trial registration:**

Trial Registration Number: NCT06124586 (Registration Date: 2023–08-2).

## Background

Diabetic foot syndrome (DFS) refers to a spectrum of foot pathologies in persons with diabetes, including microvascular and macrovascular disorders. Factors such as distal symmetric polyneuropathy (DSPN), peripheral artery disease (PAD), and immune system impairment increase the risk of soft tissue injuries and infections in diabetic foot ulcers (DFU) [1]. Infected DFU are the leading cause of diabetes-related hospital admissions [2]. The lifetime risk of developing a DFU is reported to be 19–34%, with infections occurring in 50–60% of ulcers [3]. Approximately 20% of diabetic foot infections (DFI) result in lower-extremity amputations, accounting for 44% of minor and 18% of major amputations [4]. Osteomyelitis occurs in 44–68% of hospitalised persons with DFI and is the leading cause of amputation [5].

PAD, characterised by lower-limb artery stenosis, is a critical complication of type 2 diabetes (T2D) and affects more than 50% of persons with DFS, significantly increasing the risk of amputation [6]. Similarly, microcirculatory disorders are major contributors to non-healing foot ulcers [7]. Independent of traditional risk factors, the presence of microvascular disease alone increases the risk of amputation and synergistically increases the risk in persons with PAD [8]. DSPN and impaired microcirculation are closely interrelated, as nerve damage can impair blood flow regulation, leading to further ischemia and exacerbating microvascular dysfunction. Conversely, poor microcirculation can worsen nerve damage, perpetuating a cycle that accelerates the progression of both conditions. Current peripheral blood perfusion measurements (e.g., ankle-brachial index (ABI) or duplex sonography) do not capture microcirculatory disorders, especially in the context of diabetes-induced medial arterial calcification. Peripheral tissue perfusion in DFS is clinically often underestimated due to the warm and painless presentation in peripheral polyneuropathy, potentially delaying crucial vascular treatments like percutaneous transluminal angioplasty (PTA) [9]. Minimally invasive endovascular therapy, such as PTA, is beneficial for high-risk individuals with ischemic and neuropathic DFUs, offering limb preservation rates as high as 94% [10–12]. However, amputation rates remain high in this group, and there is a lack of randomised controlled trials (RCTs) investigating the effects of early PTA in persons with PAD and DFU. It remains unclear whether early revascularisation (within 48 h) after PAD diagnosis, particularly in persons with microangiopathy, can improve outcomes.

The wound microbiome could play a key role in DFU outcomes, with microbial dysbiosis driving infection, worsening ulceration and ultimately resulting in major amputations or sepsis [13]. The beta-diversity of microbial samples can indicate differences in bacterial populations between patient samples or body sites, helping to understand variations in disease development or treatment responses [14]. Thus, the effect of PTA on changes in beta-diversity of the wound microbiome is yet not well understood [12, 15]. The microbiome composition as assessed by novel molecular (metagenomic) microbiological assessment may influence chronic infection and wound healing [16]. Current guidelines of the International Working Group on the Diabetic Foot (IWGDF) call for studies that provide new insights into these interactions [17]. PTA may alter the wound environment by enhancing oxygenation, potentially influencing the microbiome. However, restored blood flow might also lead to systemic changes, such as increased cytokine release or microbial translocation into the bloodstream [18, 19]. The role of molecular microbiological testing in guiding antimicrobial therapy and enhancing DFU outcomes requires further investigation [17]. Cell-free microbial DNA, released from infected tissues, is gaining attention as a potential biomarker for infection monitoring post-PTA [18, 19]. Results from this monocentric RCT study could guide the rational use of antibiotics and improve infection control following endovascular interventions in DFU persons. Biomarkers, such as endothelial proteins and apoptotic markers, may help diagnose ischemic DFU and predict outcomes, although their clinical utility remains unclear [20–22].

This RCT will assess whether PTA within 48 h improves wound healing and reduces complications in participants with chronic ischemic DFUs over a 12-month period compared to standard care. The primary endpoint is the effect of early PTA on wound healing. Secondary endpoints include the combined occurrence of major adverse limb events (MALE), which serve as limb-specific outcomes, and major adverse cardiac events (MACE), which are considered safety-related secondary outcomes. Additional secondary endpoints include time to complete wound healing, major (above-ankle) amputation rate, and the need for new revascularization. Explanatory objectives are changes in wound microbiome composition and local microcirculation. The findings will inform the timing of PTA in managing DFS and help tailor more personalised and effective interventions for T2D persons. The study will also explore whether the baseline wound microbiome can predict healing outcomes.

## Methods/design

### Study design and randomisation protocol

The PTA-DFS Study is a prospective, monocentric, RCT including participants with T2D and ischemic DFU. Ethics approval was obtained from the local ethics committee (Heinrich Heine University, Düsseldorf, Germany) in accordance with the Declaration of Helsinki (#2022–2291). The PTA-DFS study is registered at www.clinicaltrials.gov (NCT06124586; Registration Date: 2023–08-2).

Participants will be randomly assigned in a 1:1 ratio to either the early PTA group (‘immediate PTA‘ within 48 h after leg artery stenosis (LAS) diagnosis or the elective PTA group (standard of care with PTA within 6 weeks) at the academic hospital of the Heinrich Heine University Düsseldorf, Germany, with inclusion of 200 participants within 5 years. All study participants included in the study will provide informed consent. Although the study is unblinded for both participants and physicians due to practical feasibility, blinding will be applied during the data analysis phase, with separate analysis of primary and secondary endpoints by interventionists (physicians or clinicians) and data analysts to minimise bias. After the data is unblinded, all results will be reviewed collectively. This approach aims to ensure more accurate and unbiased analysis of the primary and secondary endpoints.

Randomisation is performed using a computer-generated sequence with block randomisation to ensure unbiased allocation. The block size will be specified in advance, using blocks of 4 or 6, to maintain balance across groups throughout the study. Both groups receive standard care, with the only difference being the timing of the PTA procedure. The study is structured to ensure that, apart from the timing of PTA, all other aspects of care are consistent across both groups. All subsequent laboratory analyses will be performed in a blinded manner, without knowledge of randomisation. Aside from the timing of PTA, baseline and follow-up examinations will be conducted uniformly across both groups. To optimise recruitment efficiency, training sessions will be held with the interventional angiology staff and the diabetic foot care team, ensuring participants and staff are fully informed about study-related procedures and analyses during their state-of-the-art clinical care.

**Fig. 1:**
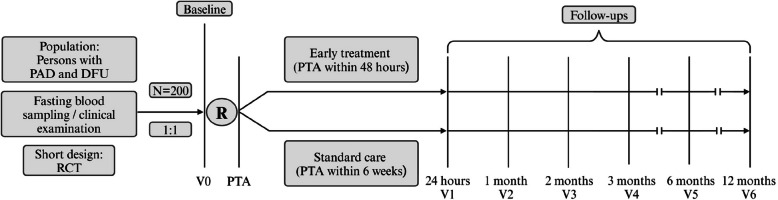
Timeline. The figure shows follow-up visits at respective time points during the study period. The specified times of the respective follow-up examinations refer to the time after the PTA. PTA = Percutaneous transluminal angioplasty, PAD = Peripheral arterial disease, T2D = Type 2 diabetes, DFU = Diabetic foot ulcer, RCT = Randomised controlled trial

As illustrated in the study flow (Fig. 1), data will be collected at baseline recruitment (V0), during the index hospitalisation (V1; PTA and 24 h thereafter), and at follow-up visits at 1 (V2), 2 (V3), 3 (V4), 6 (V5), and 12 months (V6) post-PTA. If complications in wound healing occur in the elective group, crossover to the ‘immediate‘ PTA group will be implemented. Each patient will receive guideline-directed therapy for DFU, monitored and documented by specialised staff during clinical follow-ups and all study visits. Table 1 outlines the key analyses and corresponding time points throughout the study.

**Table 1:** Study protocol. Table showing most relevant analyses and respective time points during the study period

Action								
**Visit (V)**	**Baseline**	**PTA**	**Follow-ups**					
	**V0**		**V1**	**V2**	**V3**	**V4**	**V5**	**V 6**
Time Point	0	0	0	1	2	3	6	12
Enrolment								
Eligibility Screen	X							
Informed Consent	X							
Randomisation	X							
Pseudonymisation	X							
Intervention		X						
Assessments								
Doppler/Duplex ultrasound	X		X	X	X	X	X	X
Segmental pulse oscillography	X		X	X	X	X	X	X
DSNP Screening (NDS, NSS)	X							
AI-based wound area documentation	X		X	X	X	X	X	X
Wound tissue oxygenation (NIRS)	X		X	X	X	X	X	X
AI-based retinal imaging	X							
TTE	X							
Fasting blood Sampling & Biobanking	X		X	X	X	X	X	X
Wound Swabbing & Biobanking	X		X	X	X	X	X	X
Wound Sampling & Biobanking	X		X	X	X	X	X	X
Urine Measurement	X							
Questionnaires	X		X	X	X	X	X	X
Outcome Assessment								
Primary Endpoint (wound healing)			X	X	X	X	X	X
Secondary Endpoint (MACE/MALE)			X	X	X	X	X	X

*DSPN* Distal symmetric polyneuropathy, *NDS* Neuropathy Disability Score, *NSS* Neuropathy Symptom Score, *AI* Artificial intelligence, *NIRS* Near-Infrared Spectroscopy, *TTE* Transthoracic Echocardiogram, *PTA* Percutaneous transluminal angioplasty, *MALE* Major adverse limb events, *MACE* Major adverse cardiac events

**Fig. 2:**
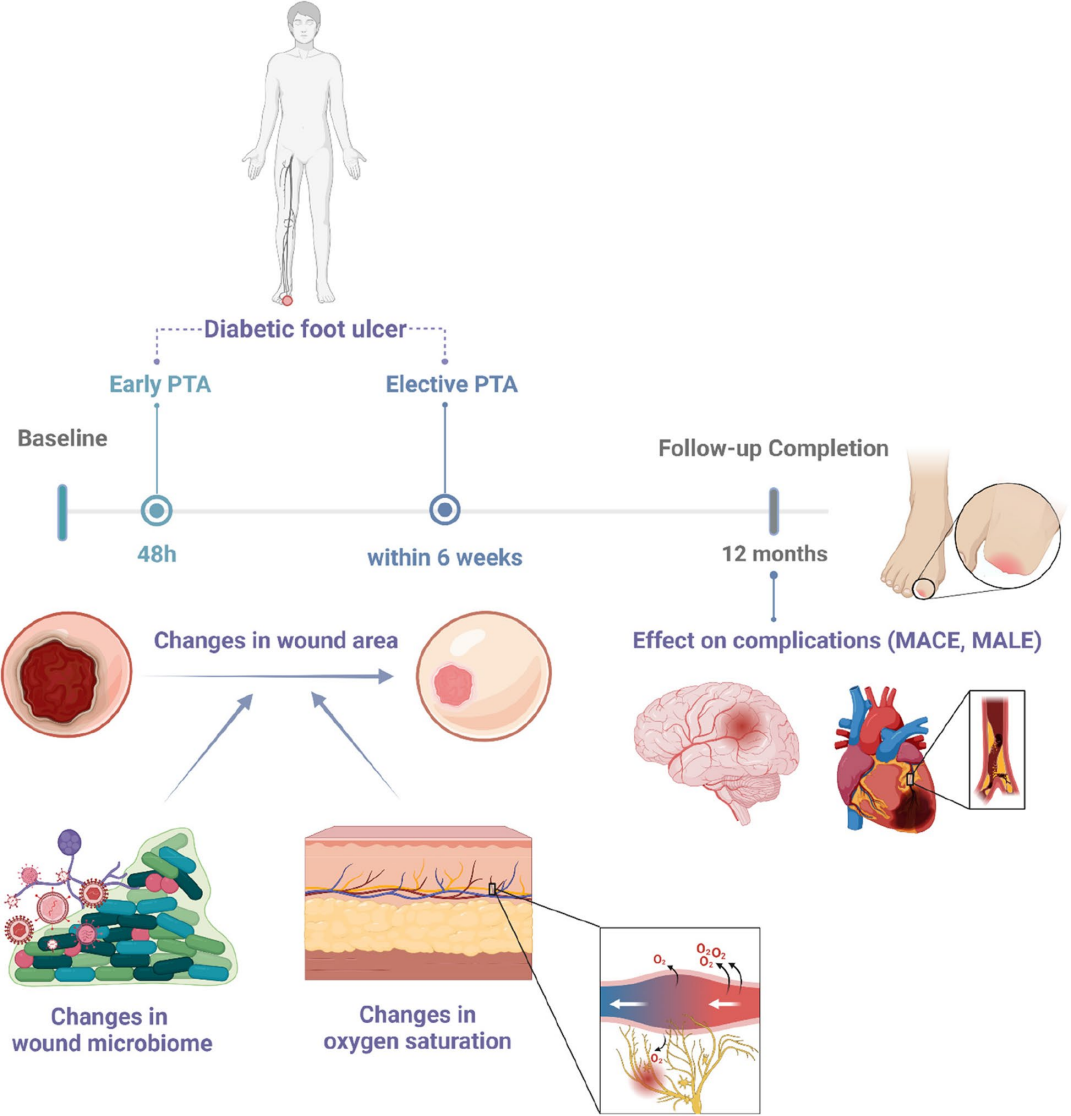
Graphical abstract. Figure illustrating the primary and secondary outcomes. The proposed study seeks to evaluate whether early PTA (‘immediate treatment‘ within 48 h) confers benefits over the ‘standard of care‘ elective approach (‘elective PTA‘) in terms of wound healing, and major adverse limb events (MALE) and major adverse cardiac events (MACE), time to complete wound healing, major amputation rate and the need for new revascularization over a follow-up period of one year. Additionally, the study will assess changes in beta-diversity of the wound microbiome and local oxygen saturation and its effects on wound healing

The primary endpoint of the PTA-DFS Study is to assess the effect of early PTA on wound healing in T2D participants and chronic ischemic DFU. Wound healing is measured by the reduction of 3D wound area, assessed using clinical grading and 3D-camera analysis with AI-based evaluation of wound length, width, and depth. The secondary endpoint is the combined incidence of MALE and MACE post-angioplasty between early and elective PTA treatment, time to complete wound healing, major amputation rate and the need for new revascularization. Explanatory variables for the primary endpoint are differences in quantitative shifts in bacterial taxa with different oxygen requirements (aerobes, anaerobes) (Fig. 2). These shifts post-PTA will be analysed using appropriate statistical methods, such as Generalised Linear Mixed Models (GLMMs), accounting for treatment group (early PTA vs. elective PTA), bacterial taxa, and time, with random effects to adjust for individual variability. Another explanatory variable for wound healing is the change in oxygen saturation in the wound environment, measured by multispectral near-infrared spectroscopy (NIRS). These outcomes will be analysed using Cox proportional hazard models using time-to-event outcome for MALE/MACE events, adjusting for baseline covariates to evaluate the effectiveness of early PTA compared to standard treatment.

### Research facilities

The Department of Endocrinology and Diabetology and Division of Cardiology, Pulmonology and Vascular Medicine have broad experiences with all clinical and laboratory methods planned for this study. The principal investigators (PI) of the PTA-DFS study are working as specialists in internal medicine, endocrinology, diabetology, angiology and cardiology in both the diabetes foot clinic and the diagnostic and interventional angiology. The clinical research unit at the Institutes provides facilities for all measurements related to this study. The laboratory facilities and corresponding protocols are well-established and routinely used. The associated research unit, the German Diabetes Center (DDZ), offers additional advantages as Germany’s leading diabetes research institution and an internationally renowned facility. It allows for further, yet unspecified, analyses at the cellular level or in mouse models for translational research. All analyses were conducted within a single-center framework (University Hospital Düsseldorf), involving various in-house departments and affiliated specialized centers, all adhering to harmonized workflows and standardized operating protocols to ensure methodological consistency and centralized quality control.

### Main exclusion criteria

- Acute leg ischemia (sudden onset, sensorimotor deficits, pale extremity, pain, loss of pulse, and shock)
- Type 1 diabetes or other forms of diabetes than T2D
- Minors or individuals incapable of giving consent
- Pregnant or breastfeeding women
- Treatment with certain drugs (immunosuppressive therapy, immunomodulators, chemotherapy, antibiotic therapy < 2 weeks before intervention for the microbiome measures)
- Diseases of the pancreas
- Severe neurological or psychiatric disease
- Known presence of malignant tumor disease within the past 5 years
- Participation in other interventional trials and receipt of investigational medication within the last 30 days
- Blood or plasma donation within the previous 3 months

### Study protocol, data, and biosample handling

Table 1 outlines key analyses and time points throughout the study. Screening and initial informed consent will be conducted by physicians in the angiology and diabetic foot clinic during outpatient visits at enrolment. A physician will verbally explain the study's objectives, methods, potential benefits, and risks before obtaining written informed consent. The complete medical history of the patient is recorded, including current and previous medications. Additionally, information on lifestyle factors (smoking, alcohol consumption, diet, physical activity, mental health, symptom severity, pain, self-management, diabetes distress, erectile dysfunction, and diabetes burden) as well as social status is collected. Participants undergo physical examination and anthropometric measurements, as well as wound swabs and sampling of deep tissue material from the wound bed for microbiome DNA sequencing. Additional proteomic and metabolomic analyses of the wound biofilm are also planned. Results will be disseminated via presentations at national and international scientific conferences and publications in peer-reviewed journals. We will engage with professional societies and patient organisations to facilitate translation into clinical practice. Participants will be offered a plain-language summary of the study results upon request. The protocol, statistical analysis plan, and de-identified analysis code will be deposited in an institutional repository upon publication. De-identified participant-level data will be made available upon reasonable request following appropriate data-sharing agreements and ethics approvals.

### Biosample collection and storage

Ulcer samples will be obtained using the standardised ‘Levine‘ technique for wound swabs and débridement material collection at baseline and follow-up visits [23]. Serial fasting blood samples will be collected for biobank storage, with consent obtained for subsequent genetic analysis. All biosamples will be preserved at −80 °C until analysis. Biosample collection, processing, and storage follow established Standard Operating Procedures (SOPs). Access to biosamples is regulated by a formal request process, reviewed by the PTA-DFS Study PIs, ensuring ethical compliance. A separate ethical approval for the CARDIAMET biobank (#2023–2525) guarantees adherence to national and international standards, ensuring sample integrity and participant confidentiality.

### Data management and security

Patient data will be securely entered into an electronic database using Research Electronic Data Capture (REDCap), fully compliant with the General Data Protection Regulation. Each participant is pseudonymised with a unique screening identifier and assigned a final study identifier at inclusion. These identifiers are used throughout data collection, storage, and reporting to protect confidentiality. Access to study data follows the same formal request process as biosamples, reviewed by the PTA-DFS Study PIs to ensure ethical and data protection compliance.

### Questionnaires

As part of the study protocol, standardised questionnaires are administered at multiple time points to comprehensively assess psychological status, lifestyle factors, comorbidities, and quality of life. At visits V0, V2, V3, V4, V5, and V6, we collect data on depressive symptoms (using PHQ-9, WHO-5, or ADS-L), diabetes distress, concomitant medication, and the presence or development of comorbidities. At visits V0, V4, and V6, additional assessments include physical activity (via the Baecke Index), smoking habits, dietary behavior (using the Food Frequency Questionnaire), mental health status, quality of life (assessed with the SF-36 questionnaire), and neuropathic symptoms (Neuropathy Symptom Score, NSS). These longitudinal assessments allow for a detailed evaluation of psychosocial and behavioral determinants as well as diabetes-related complications throughout the course of the study.

### Laboratory assessments

Venous blood samples in fasting state are processed immediately under standardised conditions, with comprehensive analyses conducted at the Central Laboratory of the University Hospital Düsseldorf as described [24]. Measurements include electrolytes, renal (creatinine, urea, cystatin C), liver function (ALT, AST, GGT, alkaline phosphatase, bilirubin), lipid profiles (cholesterol, triglycerides, HDL, LDL, Lp(a)), and glucose metabolism markers (HbA1c, insulin, glucose, C-peptide) to assess surrogates of whole-body insulin sensitivity, secretion (e. g. Homeostasis Model Assessment 2) and adipose tissue insulin resistance index [25]. Cardiovascular markers of myocardial damage, cardiac stress, and associated anaerobic metabolism (Troponin T hs, CK, CK-MB, NT-proBNP, lactate), markers of inflammations (CRP, procalcitonin, IL-6), diabetes autoantibodies (glutamate decarboxylase-, tyrosine phosphatase-, islet cell-, and zinc transporter-8-antibodies), erythropoiesis (erythropoietin, folic acid, vitamin B12, and reticulocytes) and iron metabolism (ferritin, transferrin, saturation, soluble transferrin receptor) are also measured. Thyroid function (TSH, free T4, free T3), bone health (25-hydoxy-vitamin D), blood coagulation (Quick, qPTT, fibrinogen, free haemoglobin), and haematological status (complete blood count) are evaluated. Anaemia measures include parameters of red blood cells turnover, formation, (dys)function, and haemolysis. Additionally, lactate and anaemia markers, including red blood cell function and iron status, are assessed to evaluate hypoxia and haemolysis.

### Assessment of microvascular complications

DSPN is often prevalent in DFS and assessed using the Neuropathy Disability Score (NDS), based on the modified Toronto Consensus criteria [26]. An NDS score ≥ 3 points is considered pathological [27, 28]. AI-based fundus photography is performed using a non-mydriatic camera (DRS Plus, Centervue®, Padua, Italy) to screen for diabetic retinopathy and macular degeneration [29, 30]. Utilizing confocal imaging technology, the camera provides high-quality retinal images without pupil dilation [31]. Additional complications, such as diabetic kidney disease, are assessed through urinary albumin levels (> 20 mg/l) and estimated glomerular filtration rate based on cystatin C and creatinine levels.

### Vascular and haemodynamic evaluation

At each clinical visit, haemodynamic parameters are measured, including blood pressure (in both arms after 10 min of rest) and heart rate. Comprehensive vascular evaluations include duplex/Doppler ultrasound of leg arteries, ABI assessments [32] and segmental pulse oscillography to measure perfusion pressure independent of media sclerosis often prevalent in diabetes [33]. In this study, haemodynamically relevant LAS refers to ≥ 50% narrowing of relevant arteries, confirmed by duplex ultrasound and/or an ABI < 0.9, and accompanied by clinical indicators of ischemia such as rest pain or ulceration. Peripheral artery anatomy, revascularised vessel patency, stenosis degree via peak flow velocity, and intimal hyperplasia are assessed using duplex ultrasound [34]. Local microcirculation is measured using near-infrared spectroscopy (NIRS) (SnapshotNIR, Kent Imaging, Calgary, Canada), which provides a non-invasive assessment of oxygen saturation in the affected tissues [34]. During the PTA, liquid biopsies are collected before and after significant vascular occlusions visualised by digital subtraction angiography to evaluate lactate levels for hypoxia severity as well as other biochemical and genetic markers. Of note, in cases of severe renal insufficiency, carbon dioxide angiography may be employed for selective angiography.

### Imaging and ulcer evaluation

Wound documentation includes a 3D camera with an AI-based wound analysis system (Curevision®, Munich, Germany) to objectively assess wound healing progress. The system analyses wound dimensions (length, width, area and depth), wound tissue (granulation, fibrin, and necrosis) in the wound bed, and provides consistent, non-operator-dependent data [35]. Wound documentation will be conducted at each follow-up visit until wound closure is confirmed. Tissue involvement, including possible osteomyelitis, is investigated by X-ray, computed tomography or magnetic resonance imaging if clinically indicated.

### Outcome monitoring

Over the 12-month follow-up period, MALE and MACE are monitored through patient interviews, focusing on vascular events (cardiac, cerebral, peripheral), amputations, infections, sepsis, hospitalisations, and all-cause mortality. For participants unable to attend on-site visits, telephone interviews are conducted to assess complications, including cardiovascular or all-cause mortality.

### Study organisation and leadership

The PTA-DFS Study is overseen by a dedicated Steering Committee responsible for the scientific aspects, study protocol adherence, and overall management. The study operates under the guidance of the Clinical Trial Unit at the University Hospital of Düsseldorf, following SOPs and ensuring Good Clinical Practice compliance. The Trial Management Committee conducts weekly meetings to manage day-to-day operations, while the Steering Committee convenes annually to evaluate study progress. A Data Monitoring Committee, including the data manager and Trial Management Committee members, oversees data quality and integrity, generating weekly reports to ensure accurate data collection and handling. AI-based tools were used solely for minor grammar and language editing; all scientific content was created and reviewed by the authors.

### Statistical methods and power calculation

The primary endpoint focuses on the difference in changes of wound area (delta wound area at first - last visit) in T2D participants after early PTA compared to individuals with standard PTA. For the primary—change in wound area after early PTA—and secondary endpoint—combined incidence of MALE and MACE—a power calculation was performed. Assuming a medium effect size (Cohen's d = 0.4), corresponding to 0.4 standard deviations of the difference in delta wound area and difference in time-to-event from baseline to last visit between groups, a sample size of n = 100 per group (total n = 200) is required to achieve 80% power at alpha = 0.05.

One explanatory variable evaluates the effect of early PTA on wound microbiome dynamics. This objective aims to measure the beta-diversity changes of the wound microbiome, assessed via whole-genome sequencing and analysed using Bray–Curtis distances. In detail, taxonomic profiles from whole-genome sequencing of the wound microbiome will be generated using bioinformatics scripts. The sequencing data, produced via the Oxford Nanopore platform [36] will be analysed with Kraken2 [37] and minimap2 [38] based on a custom database including protozoan, fungal, and viral sequences. Visualisations for comparing metagenomic composition across taxonomic levels will be generated with custom scripts. Principal coordinate analysis based on Bray–Curtis distances will assess sample clustering, with the entire workflow provided as a reproducible Snakemake pipeline [39]. Effect sizes are based on expected changes in Bray–Curtis distances for beta-diversity. Nominal variables will be compared using the Chi-Square Test and Fisher's Exact Test where appropriate. Relationships between variables (e. g., changes in wound microbiome or local microcirculation and wound healing or complications) will be assessed using partial Pearson or Spearman correlation coefficients, with adjustments for confounders and multiple testing via the Bonferroni or Benjamini–Hochberg method. The standardised mean difference (Cohen's d) will be used for power analyses due to the lack of preliminary data on changes of ulcer area and combined incidence of MALE/MACE after early PTA. Changes in beta-diversity of the wound microbiome, will be analysed using Generalised Linear Mixed models with fixed effects including treatment group, bacterial taxa, time, and random effects to account for patient variability. Interaction terms will assess differential effects between treatment groups. The secondary endpoint includes combined MALE and MACE incidence post-angioplasty. Oxygen saturation in wound environment is measured as another explanatory outcome potentially linked to wound healing. Additional outcomes will include an evaluation of risk factors such as comorbidities (severity of anaemia and stages of chronic kidney disease), severity of insulin resistance, DSPN, the duration of hospital stay and interactions with medications, including antibiotics and antithrombotic therapies. Risk endpoints include the incidence of MALE, progression of diabetic complications (e.g., neuropathy), and hospital stay duration. These will be analysed using survival analysis methods and logistic regression to evaluate risk/benefit ratios. Multiplicity will be addressed as follows: The primary outcome will be analysed at a two-sided significance level of 0.05 without adjustment. For the prespecified family of confirmatory secondary outcomes, the family-wise error rate will be controlled at 0.05 using the Holm–Bonferroni procedure. For these outcomes, raw and adjusted p-values as well as corresponding adjusted 95% confidence intervals will be reported. Exploratory outcomes will not be adjusted for multiplicity and will be clearly identified as exploratory. Safety outcomes will be reported descriptively. The interaction effects of antibiotics, PTA timing, and concomitant antithrombotic treatments on both primary and secondary endpoints will be assessed using logistic regression models and subgroup analyses. Time-to-event clinical endpoints will be analysed with Kaplan–Meier survival curves, while covariate adjustments will be incorporated using Cox proportional hazards models. Correlation analyses (partial Pearson or Spearman coefficients) will account for confounders and include multiple comparison corrections via Bonferroni or Benjamini–Hochberg adjustments. Categorical variables will be compared using Chi-Square or Fisher's Exact Tests, while continuous variables will be analysed with Wilcoxon rank-sum tests or Student's t-tests, as appropriate. Two-tailed p-values below 0.05 will be considered statistically significant. Missing data will be summarised by variable, time point, and treatment arm, and patterns of missingness will be described graphically and statistically. The primary analysis will employ multiple imputation by chained equations (MICE; m = 20 imputations) under the assumption of missing at random (MAR). The imputation model will be specified to be congenial with the planned analysis and will include treatment arm, relevant baseline covariates (e. g., age, sex, ulcer duration, ulcer size, Wagner grade, HbA1c, infection status), and outcome variables. Continuous variables will be imputed using predictive mean matching, binary variables will be analysed via logistic regression, and ordinal variables will be analysed using proportional-odds models. Estimates will be combined using Rubin’s rules. Sensitivity analyses will include (i) complete-case analysis, (ii) best–worst and worst–best case scenarios, and (iii) a delta-adjusted pattern-mixture model to assess departures from MAR and exploring missing not at random (MNAR). All statistical analyses will be performed using the R Version 4.4 and SPSS for Windows 23.0 (SPSS Inc., Chicago, IL, USA). Figures are created with GraphPad Prism, Version 7.01 (GraphPad Software, Inc., La Jolla, CA, USA) or R, and Graphical abstracts using BioRender.com.

## Discussion

This study investigates the effect of an early revascularisation timing on changes in wound area as well as clinical outcomes in individuals with chronic-ischemic DFU in T2D. Conducted among consecutively enrolled and prospectively followed participants treated by a multidisciplinary foot team, this is the first RCT to examine the impact of revascularisation timing on wound healing and potentially healing-associated explanatory factors such as wound microbial dynamics and changes in local microcirculation in individuals with PAD and DFU in T2D.

### Recommendations for Timing of Endovascular Therapy in Chronic-Ischemic DFU

The selected time frame for early treatment (within 48 h) was conceptually adapted from the ESC guidelines on non–ST-elevation acute coronary syndrome (NSTE-ACS), which recommend an early invasive approach within 24–72 h to prevent ischemic progression and improve outcomes. While the pathophysiology of coronary artery disease and PAD differs, the underlying principle—namely, that timely reperfusion limits tissue damage—can be applied to the context of critical limb ischemia in patients with DFS. This is supported by an observational study, which indicated that a shorter time to revascularisation (within 8 weeks) is correlated with a higher likelihood of healing in ischemic foot ulcers and limb salvage rates [40]. The time frame for follow-up visits with every 4 week examinations over 3 month is based on a post-hoc analysis suggesting that a four-week evaluation period assessing the percent change in DFU area is a robust predictor of healing at 12 weeks [41].

Limited information exists regarding the prospective outcomes of revascularisation in T2D persons with PAD and DFU. One study showed that persons with PAD who underwent revascularisation were nearly 15 times more likely to achieve wound healing compared to those who did not, with ulcer severity influencing the healing outcome [42]. Delays in revascularisation may exacerbate hypoxic conditions, favouring anaerobic bacterial proliferation, inflammation and impairing wound healing. In addition, the 48-h window represents a clinically feasible and operationally meaningful target in modern multidisciplinary diabetic foot care. Thus, this threshold balances pathophysiological urgency with realistic logistics in specialised vascular settings.

### Effects of endovascular treatment on microcirculation and wound healing

An RCT involving participants with critical limb ischemia found that poor baseline microcirculatory skin perfusion was linked to high amputation rates after 18 months [43]. Previous studies have shown that PTA not only improves macrocirculation but also positively impacts microcirculation. Changes in wound tissue oxygen saturation measured by NIRS correlated with wound area reduction, unlike ABI, which may be unreliable in persons with diabetes with media sclerosis [34]. As a non-invasive method for measuring tissue oxygenation and blood flow in the local wound environment, NIRS analyses could serve as a complementary tool to standard clinical assessments (e.g., ABI, toe-brachial index, Doppler ultrasound, transcutaneous oxygen pressure, and angiography) for evaluating microcirculatory changes after early PTA, warranting further investigation in DFU treatment strategies.

### Impact of the wound microbiome on DFU healing

Increased presence of *Lactobacillus* species has been associated with enhanced wound healing [44]. The microbiome in DFU is also different from that of unaffected skin in persons with diabetes, with skin in these individuals showing less microbial diversity compared to those without diabetes [45]. Breaches in the skin barrier expose tissue to microbial contamination, leading to infection, delayed healing, and further tissue damage. Certain strains of *Staphylococcus aureus*, *Pseudomonas aeruginosa*, *Enterococcus* spp. and *Escherichia coli* have been linked to chronic wound infections [46–48]. Pathogens that commonly infect wounds, such as *Staphylococcus aureus* and *Pseudomonas aeruginosa* express virulence factors and form biofilms, evading immunity and resisting therapies, which complicates healing [49]. Biofilm-related genes are more abundant in non-healing DFUs, highlighting the importance of microbial functional capabilities such as biofilm production in chronic wound management [45].

### Temporal dynamics of the wound microbiome

Studies indicate that rapid changes in microbial populations correlate with faster healing and better outcomes in DFU [45, 50]. Shotgun metagenomics studies show strain-specific variations, with some strains of *Staphylococcus aureus* linked to non-healing wounds, while others, like *Alcaligenes faecalis*, may not impede healing [50]. For instance, synergistic interactions between *S. aureus* and *P. aeruginosa* may exacerbate wound infections, while certain commensals may inhibit pathogen growth [45]. Understanding these microbial dynamics opens avenues for targeted therapies, including probiotics and microbiome modulation, to promote healing in DFU.

### Clinical implications for molecular microbiological analyses and early PTA

While molecular microbiological techniques offer significant insights, their clinical application faces challenges, such as difficulty in interpreting microbial roles in healing [51–53]. These methods could identify microbial shifts linked to healing outcomes, detecting early changes that precede clinical signs of infection or impaired healing, potentially enabling more targeted interventions [54–57].

However, challenges persist in adopting these techniques clinically, including high costs, complexity, and the need for specialised equipment and trained personnel [58, 59]. For example, some molecular data may be ambiguous, such as when microbial species' roles in healing are not well understood, making rapid decision-making difficult [60]. Furthermore, variability in testing protocols and the lack of consensus on relevant microbial markers hinder the development of standardised care pathways [61].

A better understanding of microbial patterns in DFU could guide personalised treatment strategies and predict healing success, though more research is needed to establish causal links between microbiome changes and healing. This study aims to explore how microbiome alterations before and after endovascular intervention can predict wound development and complications.

## Conclusion

The PTA-DFS study will examine the effect of early versus state-of-the-art elective PTA in individuals with chronic-ischemic DFU in T2D. Prospective analyses of the early PTA, performed within 48 h of diagnosing flow-limiting chronic ischemia in DFU, will clarify its impact on wound healing outcomes, MALE and MACE over one year post-angioplasty. Additionally, this study will assess how the early PTA affect changes in beta-diversity of the wound microbiome with varying oxygen requirements, local microcirculation and if these factors are linked to wound healing. These analyses are anticipated to provide essential insights for risk assessment and prognosis in these persons. Understanding the effects of early PTA intervention on these outcomes will help us develop strategies to mitigate risks and enhance clinical outcomes for this vulnerable population. Personalised treatment approaches informed by individual timing of PTA-interventions or microbiome profiles could improve wound management and limb salvage efforts. This holistic strategy aims to advance precision medicine for persons with PAD, DFU, and T2D, ultimately striving to reduce mortality rates and enhance the quality of care for individuals facing these complex health challenges.

## Data Availability

The datasets collected and analysed during the current study are not publicly available at this time because the study is ongoing and preliminary results have not yet been published in peer-reviewed journals. However, the datasets can be obtained from the corresponding authors upon reasonable request.

## Abbreviations

ABI: Ankle-brachial index
DFI: Diabetic foot infections
DFS: Diabetic foot syndrome
DFU: Diabetic foot ulcers
DSPN: Distal symmetric polyneuropathy
ESVS: European Society for Vascular Surgery
IWGDF: International Working Group on the Diabetic Foot
NDS: Neuropathy deficient score
NIRS: Near-infrared spectroscopy
PAD: Peripheral arterial disease
PI: Principal investigators
PTA: Percutaneous transluminal angioplasty
RCT: Randomised controlled trial
REDCap: Research Electronic Data Capture
SOP: Standard Operating Procedure
SVS: Society for Vascular Surgery
T2D: Type 2 diabetes
TASC II: Trans-Atlantic Inter-Society Consensus for the Management of Peripheral Arterial Disease
